# Modelling lockdown and exit strategies for COVID-19 in Singapore

**DOI:** 10.1016/j.lanwpc.2020.100004

**Published:** 2020-08-01

**Authors:** Borame L. Dickens, Joel R. Koo, Jue Tao Lim, Minah Park, Sharon Quaye, Haoyang Sun, Yinxiaohe Sun, Rachael Pung, Annelies Wilder-Smith, Louis Yi Ann Chai, Vernon J. Lee, Alex R. Cook

**Affiliations:** a Saw Swee Hock School of Public Health, National University of Singapore and National University Health System, Singapore; b Ministry of Health, Singapore; c Department of Disease Control, London School of Hygiene and Tropical Medicine, United Kingdom; d Heidelberg Institute of Global Health, Heidelberg, Germany; e Division of Infectious Diseases, University Medicine Cluster, National University Health System, Singapore; f Department of Medicine, Yong Loo Lin School of Medicine, National University of Singapore, Singapore

**Keywords:** SARS-CoV-2, Modeling, Interventions

## Abstract

**Background:**

With at least 94 countries undergoing or exiting lockdowns for contact suppression to control the COVID-19 outbreak, sustainable and public health-driven exit strategies are required. Here we explore the impact of lockdown and exit strategies in Singapore for immediate planning.

**Methods:**

We use an agent-based model to examine the impacts of epidemic control over 480 days. A limited control baseline of case isolation and household member quarantining is used. We measure the impact of lockdown duration and start date on final infection attack sizes. We then apply a 3-month gradual exit strategy, immediately re-opening schools and easing workplace distancing measures, and compare this to long-term social distancing measures.

**Findings:**

At baseline, we estimated 815 400 total infections (21.6% of the population). Early lockdown at 5 weeks with no exit strategy averted 18 500 (2.27% of baseline averted), 21 300 (2.61%) and 22 400 (2.75%) infections for 6, 8 and 9-week lockdown durations. Using the exit strategy averted a corresponding 114 700, 121 700 and 126 000 total cases, representing 12.07–13.06% of the total epidemic size under baseline. This diminishes to 9 900–11 300 for a late 8-week start time. Long-term social distancing at 6 and 8-week durations are viable but less effective.

**Interpretation:**

Gradual release exit strategies are critical to maintain epidemic suppression under a new normal. We present final infection attack sizes assuming the ongoing importation of cases, which require preparation for a potential second epidemic wave due to ongoing epidemics elsewhere.

**Funding:**

Singapore Ministry of Health, Singapore Population Health Improvement Centre.

**Research in context**

**Evidence before this study**

Contact suppression through lockdown and enhanced social distancing measures remains the frontline defence in suppressing the ongoing COVID-19 epidemic with approximately 9.2 million cases and 477 000 deaths globally at the time of writing. With at least 94 countries undergoing or leaving lockdown, there are widespread public health concerns of case resurgence due to ongoing international case importation and the non-elimination of local cases, which parallel economic and social issues from lockdown implementation. Sustainable and public-health orientated exit strategies are therefore required. We searched PubMed from database inception to May 20, 2020, for articles using the search terms “COVID-19”, “SARS-CoV-2”, “Exit Strategies” “Lockdown” and “Control Measures”. Our search yielded three modelling papers of relevance. Two articles examined control strategies within Wuhan with one focusing on the effects of social mixing and the other on case isolation. Another estimated the efficacy of long-term social distancing in the US. We found no articles which modelled the impacts of national lockdown and exit strategies for countries within the Western Pacific outside of China.

**Added value of this study**

This study investigates the use of a sustainable and public-health driven exit strategy for COVID-19, which allows for the immediate reopening of schools and gradual easing of workplace distancing. We find that the gradual release exit strategy proposed averts a substantial proportion of cases in comparison to a no exit strategy whilst simultaneously also reducing the epidemic peak size to reduce strain on the healthcare system. Such strategies can be deployed elsewhere in the region, dependent on the timing of lockdown and epidemic stage of the country.

**Implications of all the available evidence**

The results of this study provides policy makers in Singapore and other countries with evidence that gradual release exit strategies can be implemented successfully if deployed effectively and in a timely manner. Such strategies alleviate burdens on social and economic well-being from lockdown, and maintain good healthcare provisioning with sufficient planning.

Alt-text: Unlabelled box

## Introduction

In response to the rising number of local coronavirus disease 2019 (COVID-19) cases, many countries have implemented lockdowns to restrict movement and community interactions and thereby suppress infection. Details vary [1] but measures commonly include the closure of premises such as non-essential workplaces, schools, recreational facilities and places of worship. Essential services in healthcare, transport, cleaning, food services and supply chains may remain open to sustain the economy and welfare of the population. Most lockdowns have successfully reduced the reproduction number and curtailed epidemic growth, but emerging from a lockdown prematurely without sufficient planning may result in epidemic rebounding [2], as the previous suppression of cases may leave a substantial proportion of the population susceptible to infection. Intermittent lockdowns have been proposed [3] through the cycling of initiation and cessation of these interventions—possibly until 2022—to avoid exceeding hospital capacities. Concerns exist, however, on the long-term economic disruption, negative consequences on social and mental well-being, and costly administration required to ensure compliance.

Here, in the context of lockdown and social distancing measures, we explore the impact of a gradual release exit strategy (GRES) with a rollback of social distancing interventions operating outside of trigger mechanisms from emergency intermittent lockdown measures. Health and isolation facility expansion and preparation will be required during the lockdown phase regardless of control strategy to maintain a high standard of healthcare, which can substantially reduce case fatality rates [4]. GRES can provide timelines to government agencies, services and businesses, and allow society and the economy to return to a new-normal state with continuing extensive education on the importance of safe distancing, hygiene practices and precautionary measures such as mask wearing. We assess GRES through an agent-based model of a city, modelled upon the city-state of Singapore.

The implementation of social distancing measures in Singapore has been progressive from 10 March with the rollout of gradually stricter recommendations and regulations [5], leading to the implementation of a ‘circuit breaker’ [6], or lockdown, on 7 April 2020 in response to rising autochthonous cases. Although Singapore's lockdown seemingly had no effect on several large outbreaks among foreign worker dormitories that started in early April, it appears to have arrested the epidemic growth in the general population, using school closures and workplace distancing as recognized and effective attack rate reduction measures [7,8]. Substantial education and productivity losses are expected with all children tele-learning and an estimated 80% non-essential employees working from home or unable to work [9]. In response, a 3-tier financial support package for businesses and individuals has been released to provide financial assistance for families, support networks, workers and students amounting to ∼$38.8 billion USD [10]. On 21 April 2020, the Singaporean Government announced an extension of social distancing measures to 8 weeks in total with plans to end on 1 June 2020 [11].

## Methods

We utilise the geographical, demographic and epidemiological model of Singapore for respiratory diseases (GeoDEMOS-R), an agent-based epidemic simulation model comprising of a synthetic and calibrated population where the impact of interventions can be measured. The model has been previously used to estimate the effects of early epidemic control [12] and of home versus institutional isolation of cases [13], and has been updated and expanded to assess exit strategies. In summary, we investigate the effects of early social distancing, lockdown and GRES in 480 days by:

(1) Establishing a limited control baseline with case isolation and quarantining of family members only;
(2) Measuring the impact of 6-week (early cessation), 8-week (planned) and 9-week (extended) lockdown in duration, at different start dates of 5, 6, 7 and 8 weeks, on the final infection attack size, when compared to a no-exit strategy where lockdown is immediately lifted;
(3) Estimating the effects of this lockdown with GRES which includes the immediate re-opening of schools post lockdown, due to their limited epidemic suppression impact [14], with a 3-month readjustment period. In the first 2 months, 50% of the workforce returns physically to work, followed by 1 month at 75%, before full re-opening to pre-epidemic levels; and
(4) Comparing lockdown to a no-lockdown strategy with long-term social distancing of differing start times at 5, 6, 7 and 8 weeks from the epidemic start date and durations of 2, 4, 6 and 8 weeks. During this period, 50% of the adult population is actively working, schools are closed and active social distancing is being done within the community.

### Summary of synthetic population generation

The model used is GeoDEMOS-R, an agent-based epidemiological model with a synthetic resident population that is heavily calibrated to be representative. Full details are explained elsewhere; [12] here we provide a summary. Households were constructed using 178 census tables based on a sample of 200,000 households. An heuristic search algorithm was used to create a total of 3.77 million Singaporeans with the attributes of age, ethnicity and gender. Remaining attributes were drawn randomly from the specified attribute's marginal distribution and summary tables of the population comparable to the census tables formed whose fit were assessed using Pearson's chi-squared statistic. Zero count cells were avoided by setting them to 0.1. Each attribute was fitted using a Monte Carlo swapping algorithm, which swaps two random individuals’ data until the improvement in fit becomes negligible (<0.001% improvement for 10,000 runs). The same process was used to generate partners, allocate children and create multi-generational families for 1.14 million households. Households were geolocated within discrete areas named subzones according to spatial characteristics outlined in the census, and individuals allocated workplaces and schools appropriately based on distributions of commuting time data from Singapore's Household Interview Travel Study and EZ-Link data which is a 1-month record of the majority of the population's public transport activities.

### Transmission model

In the transmission model, day and night steps exist to differentiate movement behaviour and infection likelihood between individuals within the household, school, workplace and community. During the day, workers interacted with individuals in the same workgroup, and students within their classes. These two groups also interacted with those in the wider area around their workplace and school at community rates. Individuals who were not working or studying were modelled to interact with people in the same residential community. In the night step, individuals interacted primarily within their households where children had the highest probability of contact with his or her family members.

Suppose *i* and *j* are two individuals in the synthetic population with *j* becoming infected, we denote the probability of *j* infecting *i* on day *t* in location type *g* as

(1) $$P_{ijg}\left(t\right)=\beta_{g}I\left(t-t_{j}\right).$$

Here β_*g*_ is constant for location type *g*, defined as the home, workplace or school which both individuals belong to; β_*g*_ is obtained from a contact rate study (Supplementary Table 1, 2) for different social group settings in Singapore where the contact rates serve as the likelihood for individuals to infect one another at specific group locations and the wider spatial subzone area.

Overall, the probability of individual *i* getting infected from location type *g* on day *t* is therefore given as,

(2) $$p_{G}\left(t\right)=\;\frac{1}{\left|G\right|}\sum_{j\in G}P_{ijg}\left(t\right)\;=\;\frac{\beta_{g}}{\left|G\right|}\sum_{j\in G_{t}}I\left(t-t_{j}\right).$$

Here *G* is a set of individuals of location type *g, G_t_* is the subset of all individuals who belongs to set *G* and are infectious on day *t*. We use | . | to denote the size of the set of individuals. Hence, |*G*| is the total number of people in set *G*. The number of people in set *G* that would be infected on day *t* can be denoted by a random variable *X_G_*(*t*) and

(3) $$X_{G}\left(t\right)\;∼\;Bin\left(\left|G\right|-|G_{t}\left|-|G_{r}\right|,\;\;p_{G}\left(t-1\right)\right)$$

where *G_r_* is the subset of *G* consisting of individuals that have been removed through hospitalisation and subsequent recovery. The total number of people α infected on day *t* can then be expressed by summing over all the different sets of individuals in the population,

(4) $$\alpha \left(t\right)=\;\sum_{G}X_{G}\left(t\right).$$

For each simulation, the model was run for 480 days—approximately 15 months—to estimate the number of cases by the end of April 2021; cases began entering Singapore in January 2020.

### Model parameterisation and fitting

With more epidemiological information available, model parameters that were previously based on the severe acute respiratory syndrome coronavirus (SARS-CoV) have been updated (a summary of parameters and interventions is provided in Supplementary Table 1). We assumed that the basic reproductive number (*R*_0_) for SARS-CoV-2 was 2.0 [15], the asymptomatic rate was 17.9% [16] and the incubation cumulative distribution function was modified to have a median incubation period of 4 days [17]. The *R*_0_ parameter was built with a multiplier γ, which modified the infectiousness parameter of each individual in the simulation [18]. We first simulate the initial 4 weeks for a selected γ ∈ [0, 1]. This γ has a correspondence with *r* ∈ ℝ, the value of the exponential model exp(*rt* + *b*_0_) that best fits the simulated 4-week outbreak. This value of *r* was used in the linearized form of the susceptible-exposed-infectious-removed (SEIR) model [19] to compute the corresponding *R*_0_. Through this process we obtained a corresponding value of *R*_0_ for γ. A grid search was performed on γ, with γ that corresponded closest to *R*_0_ = 2.0 being selected.

For each scenario, we had case importation based on a Poisson model with λ = 2.0. To calculate λ, case importation data was used to fit a model on the expected number of daily case importations, where λ was the model's average case importation over time.

### Interventions

For this study, we ran 100 simulations for each intervention. There was no limit to isolation capacity as we assumed that the majority of the symptomatic cases will be transferred to hospital or community isolation facilities. Our baseline scenario included the isolation of ascertained cases and home quarantine of their household members. All ascertained cases are assumed to have a 24-h delay before they are no longer infectious to the wider population to accommodate for healthcare facility visitation and testing. We additionally assumed perfect compliance of those under household quarantine as strict punitive measures are in place.

For lockdown, a harsher penalisation on the contact rate was implemented with an initial 2-week period of social distancing, followed by a 6, 8 or 9-week period where schools remained closed, and work and community contact rates are further reduced to 20%. This further reduction is to simulate essential work and economic activity still being carried out by the population during the lockdown period. The starting points were at week 5, 6, 7 and 8 of the epidemic.

For the post lockdown strategy, two scenarios were modelled. The first assumed conditions went back to a pre-epidemic state with all schools, workplaces and the community at 100% transmission. The second, labelled as the gradual release exit strategy (GRES), assumed a successive restoration of contact rates over a period of three months. In the two months directly after lockdown, schools reopen and contact levels are restored at 50%, followed by 1 month of 75% restoration of workplace and community contact levels, and then pre-epidemic contact rates at 100%. This represents a cautious and planned approach to avoid heavily abnormal contact disruption from the initial lockdown period whilst maintaining a level of epidemic suppression.

For a long-term social distancing strategy, school closure occurs and we assume 50% suppression in contact rates at workplaces and within the wider community. This measure can begin at week 5, 6, 7 and 8 where at each starting point, social distancing was implemented for 2, 4, 6 and 8 weeks in duration. After the end of the social distancing period, all schools were reopened with contacts restored to pre-intervention levels. The two weeks of social distancing pre lock-down are also assumed to follow the same contact rate reduction levels with school closure.

We present the main results using a 17.9% asymptomatic proportion, and the same analysis for 44% in the Supplementary Information (Supplementary Figs. 1–4).

## Results

### Limited control versus lockdown

**Fig. 1:**
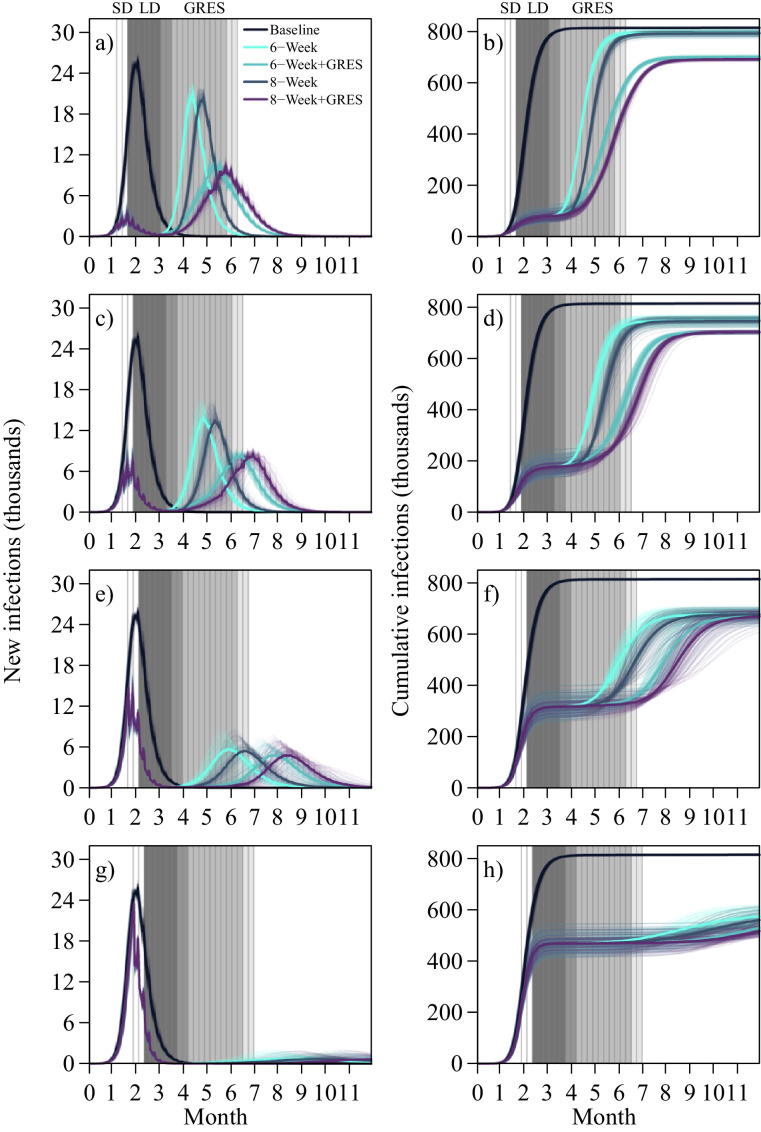
Daily new infections with lockdown measures implemented at week 5, 6, 7, 8 (a), (c), (e), (g) with corresponding cumulative values (b), (d), (f), (h) in 100 simulations. Each coloured line represents one of 100 simulations with the darker corresponding coloured line representing the median. The dark grey banded region represents 6-week lockdown (LD), medium grey the additional 2 weeks of lockdown totalling at 8 weeks, the light grey the gradual release exit strategy (GRES) for the 6-week lockdown, and the very light grey the delayed 2-weeks of GRES due to the longer implementation of the 8-week lockdown. The initial two weeks of social distancing (SD) is presented in white bands.

For the limited control baseline, the total number of infections by 480 days was 815 400 (IQR: 814 600–816 500), which represents 21.62% (21.60–21.65%) of the total population (Table 1) of which 669 500 were symptomatic (668 700–670 400). The peak of the epidemic occurred at 62 days (59–63 days) or 7 weeks with 25,800 (25 600–26 200) infected daily (Fig. 1). In comparison, the use of an early lockdown at 5 weeks with no exit strategy has a final infection attack size of 796 900 (791 800–799 300), 794 100 (790 500–798 300) and 793 000 (789 500–796 900) for early 6-week cessation, planned 8-week and prolonged 9-week lockdown respectively. The number of cases averted represents 2.27% (18 500), 2.61% (21 300) and 2.75% (22 400) of the baseline infected population. This is reduced to 591 500 (577 800–600 100), 586 600 (573 300–597 000) and 585 400 (573 200–597 500) for a late lockdown starting at week 8, with corresponding proportions of 27.46%, 28.06% and 28.21% averted.

**Table 1:** Final infection attack size of population at 480 days under 6, 8 and 9-week lockdown (LD) with and without a gradual release exit strategy (GRES) in 100 simulations. The baseline infection attack size is 815 400 (814 600–816 500) infections. The proportion of infections relative to the total population are presented in italics.

Implementation Timing	6-Week LD	6-Week LD + GRES	8-Week LD	8-week LD + GRES	9-Week LD	9-Week LD + GRES
Week 5	796 900 (791 800–799 300)	700 700 (699 000 – 702 800)	794 100 (790 500 – 798 300)	693 700 (691 000 – 695 700)	793 000 (789 500 – 796 900)	689 400 (686 800 – 692 200)
	*21.13%*	*18.58%*	*21.06%*	*18.39%*	*21.03%*	*18.28%*
Week 6	750 500 (743 100–756 200)	690 400 (688 300 – 692 000)	749 700 (741 500 – 754 300)	689 400 (686 800 – 691 500)	748 400 (741 600 – 753 800)	688 000 (685 500 – 690 200)
	*19.90%*	*18.31%*	*19.88%*	*18.28%*	*19.84%*	*18.24%*
Week 7	680 500 (669 900–686 700)	673 400 (663 800 – 679 700)	679 400 (669 300 – 686 800)	673 000 (663 700 – 680 800)	678 200 (668 800 – 686 300)	672 900 (663 800 – 679 400)
	*18.04%*	*17.86%*	*18.02%*	*17.85%*	*17.98%*	*17.84%*
Week 8	591 500 (577 800 – 600 100)	580 200 (562 000 – 595 100)	586 600 (573 300 – 597 000)	576 000 (558 600 – 591 000)	585 400 (573 200 – 597 500)	575 500 (556 600 – 592 400)
	*15.68%*	*15.38%*	*15.55%*	*15.27%*	*15.52%*	*15.26%*

A delayed post lockdown secondary peak size of 6060 (5260–6740) daily cases occurs for a 6-week lockdown duration starting at week 7, which is reduced further to 5890 (5040–6590) for an 8-week lockdown in duration (Fig. 1(e)). Whilst substantially lower than the baseline peak, the initial pre-lockdown peak is relatively high. This peak is suppressed however for a lockdown starting at week 6 (Fig. 1(c)). Although the secondary peak is greater at 14 100 (13 200–15 000) and 13 900 (12 800–14 700) daily cases respectively, it represents peak suppression throughout the epidemic in comparison to the baseline.

Lockdown start time has a larger impact than duration as relatively small ranges in the cases averted exist; 18 500–22 400, 64 900–67 000, 134 900–137 200 and 223 900–230 000 total cases at 5, 6, 7 and 8-week start times across durations of 6, 8 and 9 weeks respectively.

For an asymptomatic rate of 44%, the total number of infections by 480 days rose to 1 085 000 (1 084 000–1 086 000), representing a 24.9% increase in the infected population.

### GRES versus no exit strategy

For lockdown and GRES, early lockdown at 5-weeks had a final infection attack size of 700 700 (699 000–702 800) total cases for a 6-week lockdown, 693 700 (691 000–695 700) for 8-week lockdown, and 689 400 (686 800–692 200) for a 9-week lockdown (Table 1). The proportion averted in comparison to the baseline was 14.07%, 14.93% and 15.45%, respectively. This was reduced to 580 200 (562 000–595 100), 576 000 (558 600–591 000) and 575 500 (556 600–592 400) for an 8-week start time respectively, with a corresponding proportion of 28.06–29.42% cases averted.

Overall, when compared to the baseline, GRES averts 114 700–126 000, 125 000–127 400, 142 000–142 500, 235 200–239 900 cases at lockdown start dates of 5, 6, 7 and 8 weeks for the durations of 6, 8, and 9 weeks.

Lockdown start time has a large impact on GRES (Fig. 1 and Table 1). With early lockdown implementation at 5 weeks, when compared to no-exit strategy, GRES averts an average of 96 200 cases (12.07%) in total for a 6-week early cessation lockdown, 100 400 (12.64%) for the planned 8-week and 103 600 (13.06%) for the prolonged 9-week. At this 5-week start time, GRES also reduces the secondary peak size by 9 900 (46.92%), 10 100 (49.03%) and 10 200 (50.25%) at a ∼28-day delay respectively (Fig. 1(a)). If implemented later and close to the epidemic peak at week 8, the efficacy of GRES diminishes with 11 300 (1.91%), 10 600 (1.81%) and 9900 (1.69%) cases averted in total, with no determinable secondary peak (Fig. 1). GRES’ lessening utility with later lockdown start times is due to a large proportion of cases having already occurred in the first half of the epidemic.

Post lockdown peak sizes for a 6 week start time lockdown strategy are substantially reduced with 8740 (8580–8980), 8760 (8590–8930) and 8850 (8650–8980) daily cases for a 6, 8 and 9-week duration lockdown with GRES and 14 100 (13 200–15 000), 13 900 (12 800–14 700) and 13 800 (12 800–14 700), respectively, without (Table 1). This represents a 38.01, 36.98% and 35.87% reduction. For an earlier implementation lockdown time at 5 weeks, a 47.92%, 49.03% and 50.25% corresponding post lockdown peak reduction is observed.

Lockdown duration had a limited impact on GRES, acting as a suppressive, not a preventative, measure. When utilising the 6-week early cessation, planned 8-week lockdown and prolonged 9-week lockdown, a difference of 89 100, 94 000 and 98 300 cases can be averted across the different start dates of 5, 6, 7 and 8 weeks using GRES in comparison to lockdown alone; differing only by 9200 cases between these duration times.

At a greater asymptomatic proportion of 44% we observed similar outcomes in terms of cumulative infection numbers for lockdown implementation on week 5 and 6 (Supplementary Figs. 1 and 2) although it represented an overall accelerated epidemic. The largest difference was observed for a 6-week lockdown with GRES at week 5 where 833 600 (827 700–837 400) infections were observed in comparison to 700 700 (699 000–702 800) at an 17.9% asymptomatic rate.

### GRES vs. long term social distancing

**Fig. 2:**
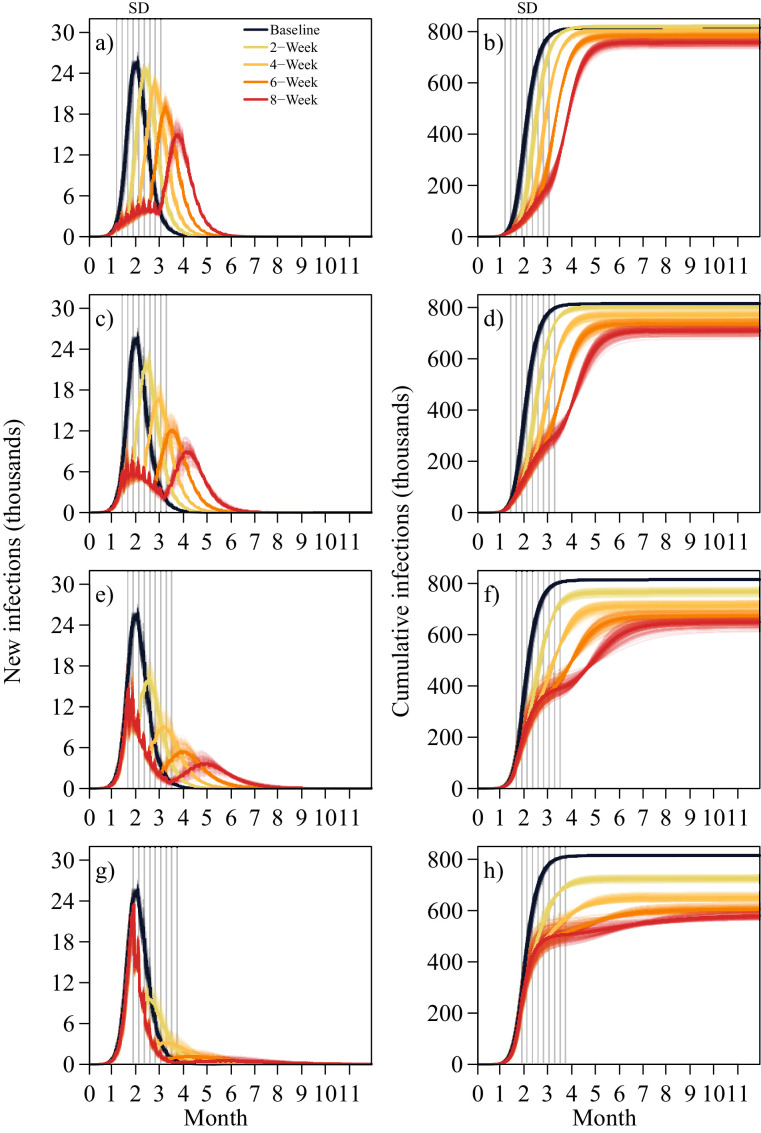
Daily new infections with social distancing measures implemented at week 5, 6, 7, 8 (a), (c), (e), (g) with corresponding cumulative values (b), (d), (f), (h) in 100 simulations. Each coloured line represents one of 100 simulations with the darker corresponding coloured line representing the median. The white bands highlight each 1-week interval of the social distancing period.

Long term social distancing at durations of 2, 4, 6 and 8-weeks cause resulting final infection attack sizes of 820 100 (818 500–821 400), 804 900 (801 300–808 000), 783 600 (779 000–786 300) and 759 000 (753 800–763 000) total cases across implementation start times of 5, 6, 7 and 8 weeks (Table 2). Post social distancing peak sizes show suppression, particularly for implementation times of week 7, although if implemented at week 6, the initial peak is also reduced (Fig. 2).

**Table 2:** Final infection attack size of population at 480 days under social distancing of 2, 4, 6, 8-week duration in 100 simulations. The proportion of infections relative to the total population are presented in italics.

Implementation Timing	2-Week	4-Week	6-Week	8-Week
Week 5	820 100 (818 500 – 821 400)	804 900 (801 300 – 808 000)	783 600 (779 000 – 786,300)	759 000 (753 800 – 763 000)
	*21.75%*	*21.34%*	*20.78%*	*20.13%*
Week 6	802 700 (799 500 – 805 000)	770 400 (765 400 – 774 900)	737 200 (729 700 – 743 000)	710 700 (705 800 – 716 800)
	*21.28%*	*20.43%*	*19.55%*	*18.85%*
Week 7	768 300 (763 300 – 771 300) *20.37%*	713 900 (706 300 – 720 800)	674 600 (666 300 – 680 400)	650 300 (642 900 – 658 600)
		*18.93%*	*17.89%*	*17.24%*
Week 8	723 900 (719 000 – 727 900)	650 900 (645 200 – 655 800)	606 700 (601 800 – 612 600)	583 200 (578 500 – 587 500)
	*19.20%*	*17.26%*	*16.09%*	*15.46%*

The secondary peaks caused by early lockdown and GRES are comparable to long-term social distancing with 50% activity in the community and the workplace, wherein 6-week interventions starting at week 7 cause a maximum of 5390 (4750–6070) cases for the former and 5490 (4710–6040) cases in the latter (Fig. 2(e)). Long term social distancing is less effective at durations of 4 weeks or lower, averting up to 1.29% of cases relative to the baseline for an implementation time at 5 weeks (Table 2). For a duration of two weeks in social distancing, a maximum of 11.22% of cases relative to the baseline can be averted if implemented close to the epidemic peak at 8 weeks.

For longer durations of 6 to 8 weeks, 3.90–6.92%, 9.59–12.84%, 17.27–20.25% and 25.59–28.48% of cases can be averted relative to the baseline for start dates of 5, 6, 7 and 8 weeks respectively. For a week 6 implementation time, 12.84% of cases can be averted with 8 weeks of social distancing relative to the baseline in comparison to 8 weeks of lockdown and GRES at 15.45%.

At an asymptomatic ratio of 44%, 2-week social distancing implemented on week 5 was the least effective, which resulted in 1 007 000 cumulative infections (995 600–1 017 000). This was the only scenario where the cumulative number of infections breached 1 million cases (Supplementary Figs. 3 and 4).

## Discussion

Critical uncertainties lie ahead on moving forward during lockdown periods in when and how to release the population back to a new normal state. Our findings suggest that earlier lockdown requires a GRES to prevent a large secondary peak, which becomes less important the later the lockdown is implemented. As Singapore entered lockdown relatively early where ∼100 cases were being reported daily, a lockdown with no exit strategy could result in up to 796 900 (791 800–799 300) total cases across 480 days, which averts 2.27% of cases in comparison to the baseline. GRES can avert up to 14% of cases for the same scenario. For lockdowns implemented at later weeks, substantial proportions of cases are averted, as observed for other epidemics [20], with a final infection attack size of 591 500 (577 800–600 100) at week 8 as an example, although this will not be viable for most countries where their healthcare capacity cannot cope with large, early epidemic peaks.

When GRES is compared with no exit strategy for a 5 and 6-week implementation start time, it results in a reduction of 8.01–13.06% in final infection attack size, and 0.78–1.91% reduction for a 7 and 8 week start time as a large proportion of cases have already occurred in the initial epidemic ramp up. GRES therefore shows good utility in populations where substantial proportions are susceptible to infection, reducing the overall outbreak size and slowing down infection spread with a greater number of transmission events prevented. This may provide opportunity to introduce prophylaxis or vaccination measures in the future, or allow the implementation of mandatory testing of all incoming travellers, which can prevent these infection events from occurring altogether as the epidemic dies out. This is especially paramount as the number of cases beyond 365 days, although considerably lower than the initial peaks, still ranges from 6.51–513.21 cases per day for GRES strategies (Supplementary Table 3a) and 4.94–90.03 for long term social distancing strategies (Supplementary Table 3b) with the ongoing risk of secondary case spread from imported cases. At the start of the epidemic however, the duration of lockdown has a limited impact on the efficacy of GRES in terms of final infection attack size, which suggests that the period should be used for epidemic preparation rather than an ongoing control method, although an extended lockdown of 8 or 9 weeks will avert more cases.

Should medical capacity permit, long term social distancing remains a viable strategy provided it is carried out for a duration of at least 6 weeks with 50% of the population working and schools closed. The total infection attack size is less for social distancing relative to lockdown with no exit strategy, but remains greater than GRES which is the most effective strategy. For overall peak size minimization, both a social distancing and GRES implementation time of 6 weeks is optimal, although if earlier lockdowns or social distancing measures have been implemented, GRES is essential for peak reduction. With a greater asymptomatic rate of 44%, the epidemic is considerably accelerated requiring earlier intervention at 4 or 5 weeks to slow infection spread. GRES remains effective at a 23.1% reduction in the final infection attack size in comparison to the baseline although these implementation dates are notably much later into the epidemic in comparison to the 17.9% asymptomatic proportion findings.

Multiple complexities exist in the framing of exit strategies to the public, including the attribution of accountability among policymakers and individuals, the acceptance of uncertain economic burdens, and need for flexibility as technologies emerge and other countries respond differently as the epidemic progresses. Lockdown and GRES can maintain public preparation, awareness and adherence which may abate if complete cessation and re-initiation of measures are introduced for extensively long periods, as observed generally for long-term therapies and lifestyle changes [21]. The strategy also partially mitigates isolation fatigue and social disruption, and should be conducted along with case finding, contact-tracing and quarantining, mass-testing in key groups such as those working with vulnerable populations, and serological surveys. With GRES in place, which will still require healthcare system ramp-up, real-time forecasting efforts can also continue to estimate appropriate intervention trigger times should another lockdown be required. Investment into the healthcare system to accommodate for the caseloads estimated in the study could avoid recurrent lockdowns, if the healthcare system can cope with the number of severe cases.

The prolonged flattening, not elimination, of the epidemic across scenarios is due to the constant influx of a conservative 2 estimated imported cases per day at the global travel hub, an estimated 17.9% (or 44%) prevalence of asymptomatic infections and inevitable future relaxation of travel restrictions to allow for influxes of short and long-term worker immigration. Additionally, containment measures and lockdowns vary widely in duration and severity between countries, [1] causing differing and delayed epidemic peaks among source countries, making lockdown a temporary protection measure against highly uncertain external epidemiological forces where delays in reactive control measures abroad or within Singapore will result in inevitable national case spread. Although seroprevalence data has yet to be released, due to the ongoing lockdown measures, a substantial proportion of the population is suspected of being susceptible to infection, which exacerbates the effects of case importation.

Ongoing concerns regarding the effects of school closure remain at the forefront of COVID-19 control policy. GRES prioritises school re-opening as clinical manifestations of children's COVID-19 appear generally less severe [22]. The societal impacts of school closure among young children, working adult parents and older children in terms of productivity and education loss are likely to be extensive and unsustainable. With continued school closure, socioeconomic inequalities will be further exacerbated, despite governmental intervention to provide intensive distance learning for all school children [23]. Overall, outside of education, schools operate as a safety net for at-risk children, providing nutritional, emotional and social support as well as vaccinations and development opportunities.

Limitations of this study include uncertainties on the current number of infections, which could not be used directly to validate the initial phase of the epidemic in the agent-based model. Parameter estimations from larger global studies were therefore used to simulate epidemics although wide ranges in observations continue to be reported for parameters such as asymptomatic rates. The model can be calibrated in the future with real time forecasting efforts and results of serology surveys to better reflect COVID-19 prevalence and incidence within Singapore. Intra workplace and school contact patterns were additionally not accounted for, which may cause greater or fewer localized contact events depending on internal grouping structures.

Further uncertainties include the current and future adherence to social distancing measures, future importation rates and spatial heterogeneities in infection rates. Ongoing epidemics in high density accommodation (Supplementary Figure 5) and their effects in the wider community were also not accounted for, which requires further investigation.

## Contributors

BLD and JRK performed the modelling and wrote the manuscript. BLD, JRK, VJL and ARC designed the intervention strategies. MP and SQ performed data collection. JTL, HS, YS, RP, AWS, LYAC, VJL and ARC critically revised the manuscript.

## Role of funding source

The study sponsors had no role in the study design, analysis, interpretation of the data or writing of the report.

## Data sharing statement

All data used within the study is publicly available. GeoDEMOS-R data is available on request.

## Declaration of Competing Interest

The authors declare no competing interests.

## Appendix Supplementary materials

Image, application 1
