# Supplementary material for: Modelling lockdown and exit strategies for COVID-19 in Singapore
Source: Lancet Reg Health West Pac. 2020 Aug 1;1:100004. doi: 10.1016/j.lanwpc.2020.100004 (PMC7395828; doi:10.1016/j.lanwpc.2020.100004)
Supplement: Supplementary file 1 [file mmc1.docx]

**Supplementary Information**

| Parameter | Value | References |
| --- | --- | --- |
| $R_{0}$ | 2.0 | 1 |
| Susceptibility | Uniform across all age groups | - |
| Incubation Period | Median 4 (IQR 3-6) | 2 |
| Asymptomatic Rate | 17.9% and 44% | 3, 4, 5 |
| Ascertainment Delay | 1 day after symptom onset | - |
| Contact Rate | From contact matrices (Table 2a, 2b, 2c and 2d), weighted by size of social group | 6,7 |
| Isolation Capacity | No maximum: will be isolated at community quarantine facilities | - |
| Isolation/Quarantine  Duration | 14 days | - |
| Quarantine Type | Household quarantine of contacts; Can only infect other household members if quarantined individual is infectious |  |
| Importation | $Importation \sim Poisson(\lambda=2)$ | - |
| Simulation Run Length | 360 days |  |
| Number of Simulations | 100 simulations for each scenario |  |
| Social Distancing Start Timings | 5, 6, 7 and 8 weeks after the start of simulation | - |
| Social Distancing Duration | 2, 4, 6 and 8 weeks of Social Distancing | - |
| Social Distancing Measures | Closure of all schools  50% reduction in workplace and community contact rates | - |
| Lockdown Start Timings | 5, 6, 7 and 8 weeks after the start of simulation | - |
| Lockdown Duration | 2 weeks of Social Distancing, followed by 6, 8 and 9 weeks of Lockdown | - |
| Lockdown Measures | Closure of all schools  80% reduction in workplace and community contact rates for the Lockdown duration | - |
| GRES Duration | 12 weeks | - |
| GRES Measures | All schools reopen  Workplace and community contact rates restored to 50% of  pre-intervention setting for the initial 8 out of 12 weeks  Workplace and community contact rates restored to 75% of  pre-intervention setting for the final 4 out of 12 weeks  Workplace and community contact rates restored to  pre-intervention setting for the remainder of the simulation | - |

Supplementary Table 1. Table of epidemiological and intervention parameters

Supplementary Tables 2a, 2b, 2c and 2d are the contact matrices taken from the POLYMOD studies^6,7^. They were used in our model to determine the contact rate of individuals across different age groups and settings (home, work, school and community).

|  | 0-4 | 5-9 | 10-14 | 15-19 | 20-24 | 25-29 | 30-34 | 35-39 | 40-44 | 45-49 | 50-54 | 55-59 | 60-64 | 65-69 | 70-74 | 75-80 |
| --- | --- | --- | --- | --- | --- | --- | --- | --- | --- | --- | --- | --- | --- | --- | --- | --- |
| 0-4 | 0.44 | 0.22 | 0.12 | 0.10 | 0.17 | 0.25 | 0.35 | 0.32 | 0.22 | 0.16 | 0.21 | 0.18 | 0.12 | 0.08 | 0.05 | 0.02 |
| 5-9 | 0.24 | 1.03 | 0.42 | 0.13 | 0.11 | 0.20 | 0.30 | 0.37 | 0.31 | 0.16 | 0.13 | 0.14 | 0.13 | 0.08 | 0.04 | 0.02 |
| 10-14 | 0.09 | 0.52 | 2.06 | 0.35 | 0.23 | 0.19 | 0.26 | 0.34 | 0.39 | 0.26 | 0.18 | 0.12 | 0.08 | 0.07 | 0.05 | 0.03 |
| 15-19 | 0.05 | 0.18 | 0.96 | 3.12 | 0.74 | 0.34 | 0.26 | 0.34 | 0.34 | 0.32 | 0.15 | 0.08 | 0.06 | 0.04 | 0.02 | 0.01 |
| 20-24 | 0.07 | 0.10 | 0.17 | 1.21 | 1.93 | 0.74 | 0.54 | 0.38 | 0.30 | 0.37 | 0.24 | 0.16 | 0.06 | 0.04 | 0.04 | 0.03 |
| 25-29 | 0.12 | 0.07 | 0.07 | 0.30 | 0.83 | 1.02 | 0.72 | 0.52 | 0.34 | 0.33 | 0.29 | 0.16 | 0.06 | 0.04 | 0.02 | 0.01 |
| 30-34 | 0.15 | 0.12 | 0.20 | 0.18 | 0.46 | 0.65 | 1.01 | 0.77 | 0.49 | 0.39 | 0.42 | 0.32 | 0.14 | 0.08 | 0.04 | 0.03 |
| 35-39 | 0.14 | 0.20 | 0.16 | 0.13 | 0.31 | 0.52 | 0.81 | 1.06 | 0.74 | 0.50 | 0.36 | 0.30 | 0.25 | 0.14 | 0.08 | 0.03 |
| 40-44 | 0.10 | 0.15 | 0.26 | 0.18 | 0.32 | 0.37 | 0.62 | 0.72 | 0.78 | 0.51 | 0.37 | 0.18 | 0.16 | 0.09 | 0.07 | 0.02 |
| 45-49 | 0.03 | 0.06 | 0.09 | 0.18 | 0.24 | 0.29 | 0.44 | 0.53 | 0.51 | 0.55 | 0.45 | 0.22 | 0.15 | 0.08 | 0.07 | 0.04 |
| 50-54 | 0.05 | 0.11 | 0.14 | 0.23 | 0.42 | 0.51 | 0.46 | 0.48 | 0.55 | 0.65 | 0.54 | 0.44 | 0.26 | 0.11 | 0.07 | 0.04 |
| 55-59 | 0.07 | 0.08 | 0.09 | 0.12 | 0.29 | 0.48 | 0.61 | 0.54 | 0.53 | 0.40 | 0.61 | 0.60 | 0.37 | 0.17 | 0.09 | 0.04 |
| 60-64 | 0.05 | 0.06 | 0.06 | 0.10 | 0.21 | 0.31 | 0.39 | 0.51 | 0.44 | 0.38 | 0.36 | 0.46 | 0.39 | 0.23 | 0.16 | 0.06 |
| 65-69 | 0.04 | 0.06 | 0.04 | 0.05 | 0.13 | 0.20 | 0.31 | 0.30 | 0.28 | 0.24 | 0.28 | 0.31 | 0.28 | 0.20 | 0.10 | 0.05 |
| 70-74 | 0.01 | 0.03 | 0.05 | 0.11 | 0.10 | 0.14 | 0.18 | 0.26 | 0.31 | 0.25 | 0.21 | 0.22 | 0.35 | 0.25 | 0.23 | 0.07 |
| 75-80 | 0.02 | 0.02 | 0.03 | 0.02 | 0.04 | 0.06 | 0.13 | 0.11 | 0.11 | 0.14 | 0.11 | 0.11 | 0.10 | 0.11 | 0.09 | 0.05 |

Supplementary Table 2a. Contact matrix for communities by age group.

|  | 0-4 | 5-9 | 10-14 | 15-19 | 20-24 | 25-29 | 30-34 | 35-39 | 40-44 | 45-49 | 50-54 | 55-59 | 60-64 | 65-69 | 70-74 | 75-80 |
| --- | --- | --- | --- | --- | --- | --- | --- | --- | --- | --- | --- | --- | --- | --- | --- | --- |
| 0-4 | 0.54 | 0.37 | 0.16 | 0.09 | 0.18 | 0.32 | 0.46 | 0.45 | 0.23 | 0.07 | 0.05 | 0.02 | 0.02 | 0.01 | 0.01 | 0.01 |
| 5-9 | 0.24 | 0.84 | 0.40 | 0.16 | 0.05 | 0.19 | 0.42 | 0.53 | 0.48 | 0.14 | 0.07 | 0.04 | 0.02 | 0.01 | 0.01 | 0.01 |
| 10-14 | 0.12 | 0.41 | 1.52 | 0.43 | 0.07 | 0.05 | 0.12 | 0.42 | 0.65 | 0.24 | 0.09 | 0.02 | 0.01 | 0.02 | 0.01 | 0.01 |
| 15-19 | 0.06 | 0.14 | 0.41 | 1.33 | 0.21 | 0.06 | 0.03 | 0.26 | 0.46 | 0.43 | 0.20 | 0.05 | 0.02 | 0.01 | 0.01 | 0.00 |
| 20-24 | 0.13 | 0.07 | 0.08 | 0.35 | 1.28 | 0.21 | 0.05 | 0.02 | 0.13 | 0.33 | 0.22 | 0.10 | 0.02 | 0.00 | 0.00 | 0.01 |
| 25-29 | 0.33 | 0.13 | 0.04 | 0.09 | 0.25 | 0.98 | 0.18 | 0.03 | 0.02 | 0.09 | 0.20 | 0.11 | 0.05 | 0.01 | 0.00 | 0.01 |
| 30-34 | 0.52 | 0.55 | 0.24 | 0.05 | 0.08 | 0.24 | 1.04 | 0.30 | 0.07 | 0.02 | 0.05 | 0.07 | 0.06 | 0.02 | 0.01 | 0.01 |
| 35-39 | 0.49 | 0.76 | 0.66 | 0.36 | 0.04 | 0.05 | 0.22 | 1.16 | 0.22 | 0.04 | 0.03 | 0.02 | 0.04 | 0.03 | 0.02 | 0.00 |
| 40-44 | 0.25 | 0.52 | 0.71 | 0.52 | 0.12 | 0.04 | 0.08 | 0.19 | 0.87 | 0.16 | 0.05 | 0.01 | 0.03 | 0.03 | 0.01 | 0.01 |
| 45-49 | 0.14 | 0.30 | 0.46 | 0.66 | 0.35 | 0.10 | 0.04 | 0.09 | 0.18 | 0.77 | 0.17 | 0.04 | 0.02 | 0.01 | 0.01 | 0.02 |
| 50-54 | 0.22 | 0.21 | 0.36 | 0.44 | 0.41 | 0.26 | 0.11 | 0.05 | 0.10 | 0.22 | 0.76 | 0.17 | 0.03 | 0.01 | 0.01 | 0.03 |
| 55-59 | 0.39 | 0.42 | 0.27 | 0.36 | 0.32 | 0.37 | 0.27 | 0.10 | 0.05 | 0.15 | 0.26 | 0.80 | 0.16 | 0.05 | 0.01 | 0.02 |
| 60-64 | 0.38 | 0.37 | 0.24 | 0.22 | 0.16 | 0.21 | 0.26 | 0.20 | 0.10 | 0.06 | 0.10 | 0.21 | 0.67 | 0.14 | 0.02 | 0.01 |
| 65-69 | 0.21 | 0.32 | 0.29 | 0.14 | 0.10 | 0.11 | 0.19 | 0.23 | 0.20 | 0.07 | 0.06 | 0.09 | 0.13 | 0.49 | 0.07 | 0.01 |
| 70-74 | 0.11 | 0.31 | 0.27 | 0.21 | 0.04 | 0.09 | 0.09 | 0.21 | 0.16 | 0.14 | 0.08 | 0.05 | 0.08 | 0.12 | 0.29 | 0.06 |
| 75-80 | 0.17 | 0.23 | 0.35 | 0.27 | 0.07 | 0.06 | 0.08 | 0.15 | 0.19 | 0.17 | 0.20 | 0.09 | 0.04 | 0.07 | 0.06 | 0.19 |

Supplementary Table 2b. Contact matrix for homes by age group.

|  | 0-4 | 5-9 | 10-14 | 15-19 | 20-24 | 25-29 | 30-34 | 35-39 | 40-44 | 45-49 | 50-54 | 55-59 | 60-64 | 65-69 | 70-74 | 75-80 |
| --- | --- | --- | --- | --- | --- | --- | --- | --- | --- | --- | --- | --- | --- | --- | --- | --- |
| 0-4 | 0.00 | 0.00 | 0.00 | 0.00 | 0.00 | 0.00 | 0.00 | 0.00 | 0.00 | 0.00 | 0.00 | 0.00 | 0.00 | 0.00 | 0.00 | 0.00 |
| 5-9 | 0.00 | 0.00 | 0.00 | 0.00 | 0.00 | 0.00 | 0.00 | 0.00 | 0.00 | 0.00 | 0.00 | 0.00 | 0.00 | 0.00 | 0.00 | 0.00 |
| 10-14 | 0.00 | 0.00 | 0.01 | 0.01 | 0.01 | 0.00 | 0.02 | 0.01 | 0.02 | 0.01 | 0.00 | 0.00 | 0.00 | 0.00 | 0.00 | 0.00 |
| 15-19 | 0.00 | 0.00 | 0.01 | 0.49 | 0.53 | 0.30 | 0.27 | 0.25 | 0.27 | 0.21 | 0.13 | 0.06 | 0.01 | 0.00 | 0.00 | 0.00 |
| 20-24 | 0.00 | 0.00 | 0.02 | 0.36 | 0.93 | 0.89 | 0.72 | 0.77 | 0.58 | 0.46 | 0.36 | 0.17 | 0.06 | 0.00 | 0.00 | 0.00 |
| 25-29 | 0.00 | 0.00 | 0.02 | 0.31 | 0.88 | 1.48 | 1.03 | 0.98 | 0.90 | 0.62 | 0.53 | 0.25 | 0.07 | 0.00 | 0.00 | 0.00 |
| 30-34 | 0.00 | 0.00 | 0.03 | 0.16 | 0.60 | 0.98 | 1.24 | 1.06 | 0.94 | 0.76 | 0.47 | 0.29 | 0.07 | 0.00 | 0.00 | 0.00 |
| 35-39 | 0.00 | 0.00 | 0.02 | 0.32 | 0.48 | 0.89 | 0.93 | 1.25 | 1.21 | 0.83 | 0.62 | 0.26 | 0.05 | 0.00 | 0.00 | 0.00 |
| 40-44 | 0.00 | 0.00 | 0.02 | 0.20 | 0.56 | 0.88 | 1.00 | 1.05 | 1.27 | 1.01 | 0.75 | 0.27 | 0.07 | 0.00 | 0.00 | 0.00 |
| 45-49 | 0.00 | 0.00 | 0.02 | 0.24 | 0.38 | 0.65 | 0.81 | 0.87 | 0.89 | 0.86 | 0.57 | 0.30 | 0.05 | 0.00 | 0.00 | 0.00 |
| 50-54 | 0.00 | 0.00 | 0.02 | 0.19 | 0.32 | 0.67 | 0.75 | 0.76 | 1.03 | 0.98 | 0.77 | 0.39 | 0.06 | 0.00 | 0.00 | 0.00 |
| 55-59 | 0.00 | 0.00 | 0.04 | 0.13 | 0.23 | 0.42 | 0.57 | 0.52 | 0.67 | 0.52 | 0.50 | 0.32 | 0.06 | 0.00 | 0.00 | 0.00 |
| 60-64 | 0.00 | 0.00 | 0.01 | 0.02 | 0.09 | 0.17 | 0.18 | 0.20 | 0.22 | 0.21 | 0.17 | 0.13 | 0.02 | 0.00 | 0.00 | 0.00 |
| 65-69 | 0.00 | 0.00 | 0.00 | 0.00 | 0.00 | 0.00 | 0.00 | 0.00 | 0.00 | 0.00 | 0.00 | 0.00 | 0.00 | 0.00 | 0.00 | 0.00 |
| 70-74 | 0.00 | 0.00 | 0.00 | 0.00 | 0.00 | 0.00 | 0.00 | 0.00 | 0.00 | 0.00 | 0.00 | 0.00 | 0.00 | 0.00 | 0.00 | 0.00 |
| 75-80 | 0.00 | 0.00 | 0.00 | 0.00 | 0.00 | 0.00 | 0.00 | 0.00 | 0.00 | 0.00 | 0.00 | 0.00 | 0.00 | 0.00 | 0.00 | 0.00 |

Supplementary Table 2c. Contact matrix for workplaces by age group.

|  | 0-4 | 5-9 | 10-14 | 15-19 | 20-24 | 25-29 | 30-34 | 35-39 | 40-44 | 45-49 | 50-54 | 55-59 | 60-64 | 65-69 | 70-74 | 75-80 |
| --- | --- | --- | --- | --- | --- | --- | --- | --- | --- | --- | --- | --- | --- | --- | --- | --- |
| 0-4 | 0.32 | 0.04 | 0.01 | 0.01 | 0.01 | 0.02 | 0.04 | 0.03 | 0.01 | 0.02 | 0.01 | 0.01 | 0.00 | 0.00 | 0.00 | 0.00 |
| 5-9 | 0.05 | 4.35 | 0.25 | 0.03 | 0.02 | 0.06 | 0.10 | 0.10 | 0.09 | 0.07 | 0.06 | 0.02 | 0.01 | 0.00 | 0.00 | 0.00 |
| 10-14 | 0.00 | 0.96 | 5.67 | 0.18 | 0.01 | 0.05 | 0.06 | 0.10 | 0.10 | 0.08 | 0.06 | 0.03 | 0.01 | 0.00 | 0.00 | 0.00 |
| 15-19 | 0.00 | 0.04 | 1.75 | 5.99 | 0.05 | 0.06 | 0.07 | 0.11 | 0.09 | 0.11 | 0.06 | 0.04 | 0.01 | 0.00 | 0.00 | 0.00 |
| 20-24 | 0.01 | 0.02 | 0.01 | 0.52 | 0.25 | 0.03 | 0.02 | 0.03 | 0.02 | 0.02 | 0.01 | 0.01 | 0.00 | 0.00 | 0.00 | 0.00 |
| 25-29 | 0.01 | 0.09 | 0.03 | 0.14 | 0.17 | 0.12 | 0.02 | 0.03 | 0.04 | 0.03 | 0.01 | 0.01 | 0.00 | 0.00 | 0.00 | 0.00 |
| 30-34 | 0.01 | 0.35 | 0.23 | 0.15 | 0.03 | 0.07 | 0.07 | 0.05 | 0.06 | 0.03 | 0.02 | 0.00 | 0.01 | 0.00 | 0.00 | 0.00 |
| 35-39 | 0.02 | 0.22 | 0.16 | 0.08 | 0.01 | 0.05 | 0.07 | 0.06 | 0.06 | 0.03 | 0.00 | 0.01 | 0.00 | 0.00 | 0.00 | 0.00 |
| 40-44 | 0.01 | 0.12 | 0.10 | 0.39 | 0.01 | 0.02 | 0.03 | 0.04 | 0.08 | 0.03 | 0.03 | 0.01 | 0.01 | 0.00 | 0.00 | 0.00 |
| 45-49 | 0.07 | 0.28 | 0.18 | 0.64 | 0.00 | 0.03 | 0.06 | 0.06 | 0.05 | 0.03 | 0.04 | 0.02 | 0.00 | 0.00 | 0.00 | 0.00 |
| 50-54 | 0.02 | 0.46 | 0.58 | 0.62 | 0.01 | 0.02 | 0.05 | 0.05 | 0.06 | 0.09 | 0.04 | 0.02 | 0.01 | 0.00 | 0.00 | 0.00 |
| 55-59 | 0.06 | 0.43 | 0.38 | 0.42 | 0.01 | 0.06 | 0.03 | 0.04 | 0.06 | 0.04 | 0.04 | 0.04 | 0.01 | 0.00 | 0.00 | 0.00 |
| 60-64 | 0.04 | 0.09 | 0.04 | 0.21 | 0.01 | 0.00 | 0.02 | 0.06 | 0.01 | 0.02 | 0.02 | 0.01 | 0.03 | 0.01 | 0.00 | 0.00 |
| 65-69 | 0.00 | 0.03 | 0.01 | 0.00 | 0.00 | 0.00 | 0.01 | 0.01 | 0.01 | 0.01 | 0.00 | 0.02 | 0.01 | 0.02 | 0.01 | 0.00 |
| 70-74 | 0.00 | 0.00 | 0.00 | 0.01 | 0.00 | 0.00 | 0.00 | 0.01 | 0.01 | 0.02 | 0.04 | 0.02 | 0.01 | 0.01 | 0.01 | 0.02 |
| 75-80 | 0.00 | 0.02 | 0.00 | 0.02 | 0.00 | 0.01 | 0.00 | 0.00 | 0.00 | 0.00 | 0.00 | 0.00 | 0.00 | 0.00 | 0.00 | 0.00 |

Supplementary Table 2d. Contact matrix for schools by age group.

| Implementation Timing | 6-Week LD | 6-Week LD + GRES | 8-Week LD | 8-week LD + GRES | 9-Week LD | 9-Week LD + GRES |
| --- | --- | --- | --- | --- | --- | --- |
| Week 5 | 5.22 | 6.51 | 5.21 | 6.78 | 5.28 | 7.00 |
| Week 6 | 5.89 | 6.78 | 5.87 | 7.00 | 5.28 | 7.01 |
| Week 7 | 8.29 | 15.49 | 8.64 | 25.86 | 9.47 | 34.12 |
| Week 8 | 124.31 | 412.0 | 217.15 | 483.21 | 257.66 | 513.21 |

Supplementary Table 3a. Average number of daily infections from day 365 to day 480 under 6, 8 and 9-week lockdown (LD) with and without a gradual release exit strategy (GRES) in 100 simulations.

| Implementation Timing | 2-Week | 4-Week | 6-Week | 8-Week |
| --- | --- | --- | --- | --- |
| Week 5 | 4.94 | 5.04 | 5.36 | 5.72 |
| Week 6 | 4.99 | 5.50 | 6.08 | 7.02 |
| Week 7 | 5.66 | 6.66 | 9.08 | 15.12 |
| Week 8 | 6.55 | 10.34 | 26.23 | 90.03 |

Supplementary Table 3b. Average number of daily infections from day 365 to day 480 under social distancing of 2, 4, 6, 8-week duration in 100 simulations.


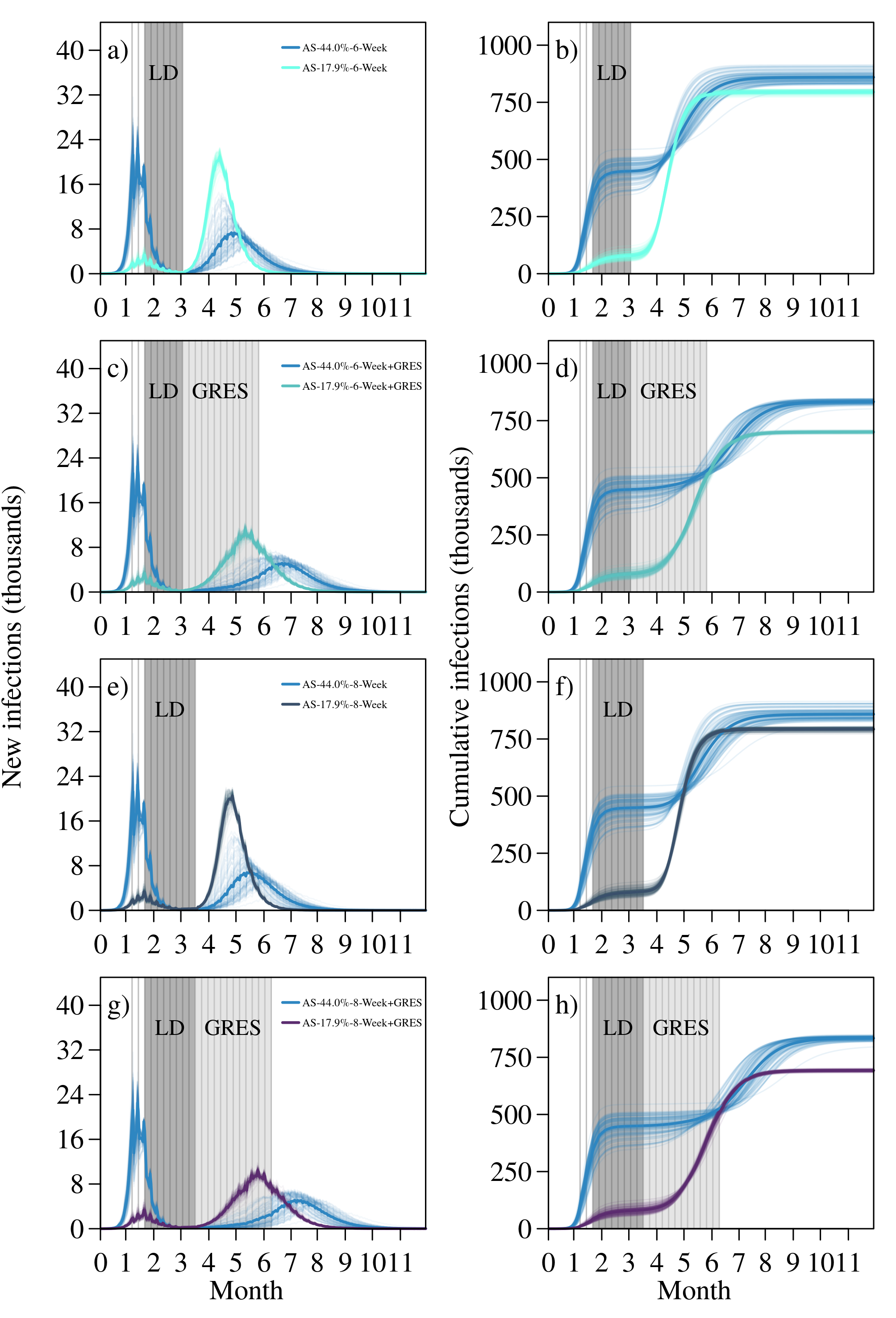


Supplementary Figure 1. Simulation runs of asymptomatic rate 17.9% and 44%, for lockdown measures implemented at week 5. Each coloured line represents one of 100 simulations with the darker corresponding coloured line representing the median. The number of daily cases (panel a, c, e, g) and cumulative cases (panel b, d, f, h) are presented. The initial white banded region represent the two initial weeks of social distancing, which occurred before the 6-week (dark grey region) lockdown (panel a, b, c, d) or 8-week lockdown (panel e, f, g, h). The subsequent light grey region (panel c, d, g, h) corresponds to the GRES period that took place over 12 weeks after the end of the respective lockdown periods.


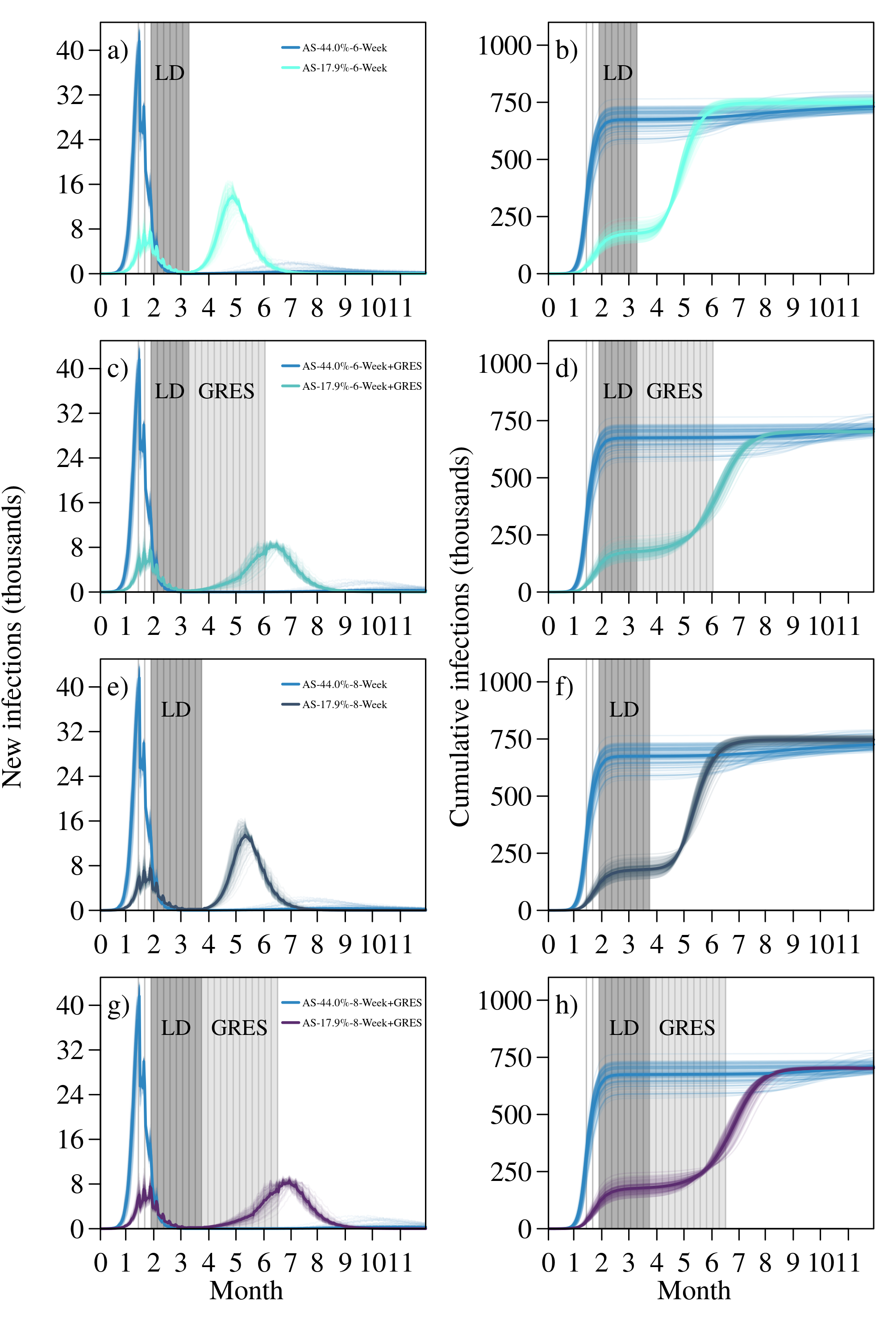


Supplementary Figure 2. Simulation runs of asymptomatic rate 17.9% and 44%, for lockdown measures implemented at week 6. Each coloured line represents one of 100 simulations with the darker corresponding coloured line representing the median. The number of daily cases (panel a, c, e, g) and cumulative cases (panel b, d, f, h) are presented. The initial white banded regions represent the two initial weeks of social distancing, which occurred before the 6-week (dark grey region) lockdown (panel a, b, c, d) or 8-week lockdown (panel e, f, g, h). The subsequent light grey region (panel c, d, g, h) corresponds to the GRES period that took place over 12 weeks after the end of the respective lockdown periods.


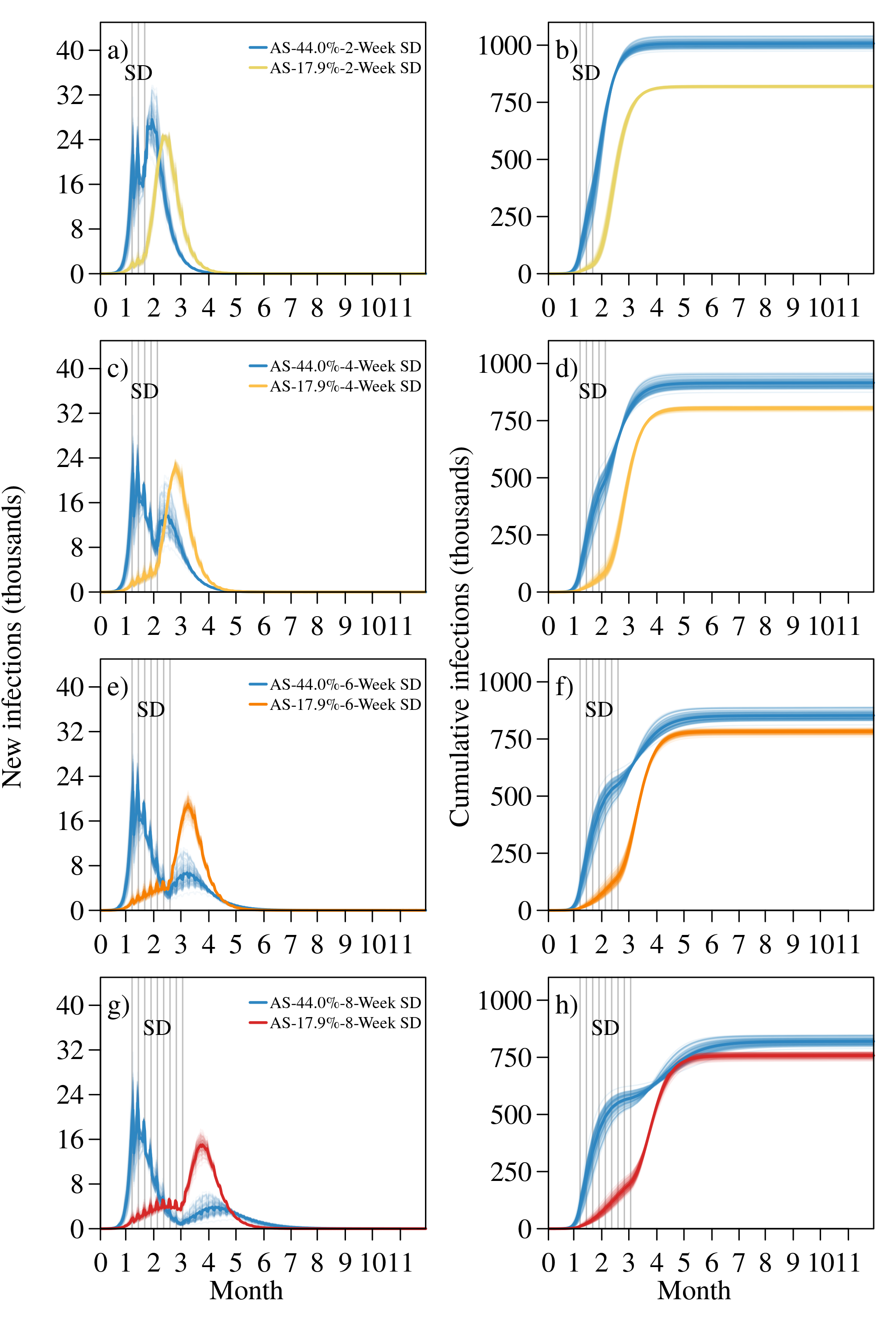


Supplementary Figure 3. Simulation runs of asymptomatic rate 17.9% and 44%, for social distancing measures implemented at week 5. Each coloured line represents one of 100 simulations with the darker corresponding coloured line representing the median. The white banded regions represent 2 weeks (panel a, b), 4 weeks (panel c, d), 6 weeks (panel e, f) or 8 weeks (panel g, h) of social distancing.


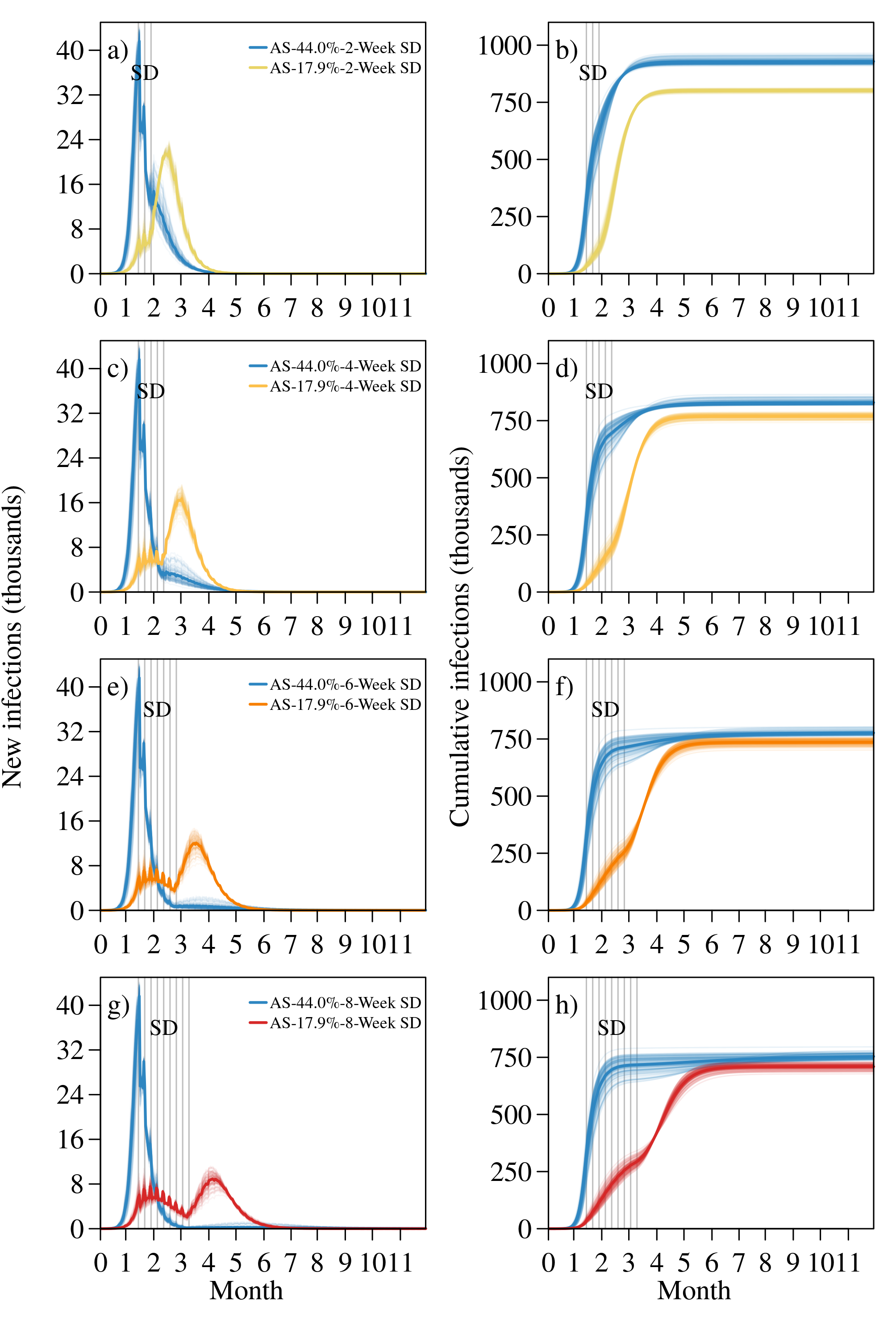


Supplementary Figure 4. Simulation runs of asymptomatic rates 17.9% and 44% for social distancing measures implemented at week 6. Each coloured line represents one of 100 simulations with the darker corresponding coloured line representing the median. The white banded regions represent 2 weeks (panel a, b), 4 weeks (panel c, d), 6 weeks (panel e, f) or 8 weeks (panel g, h) of social distancing.


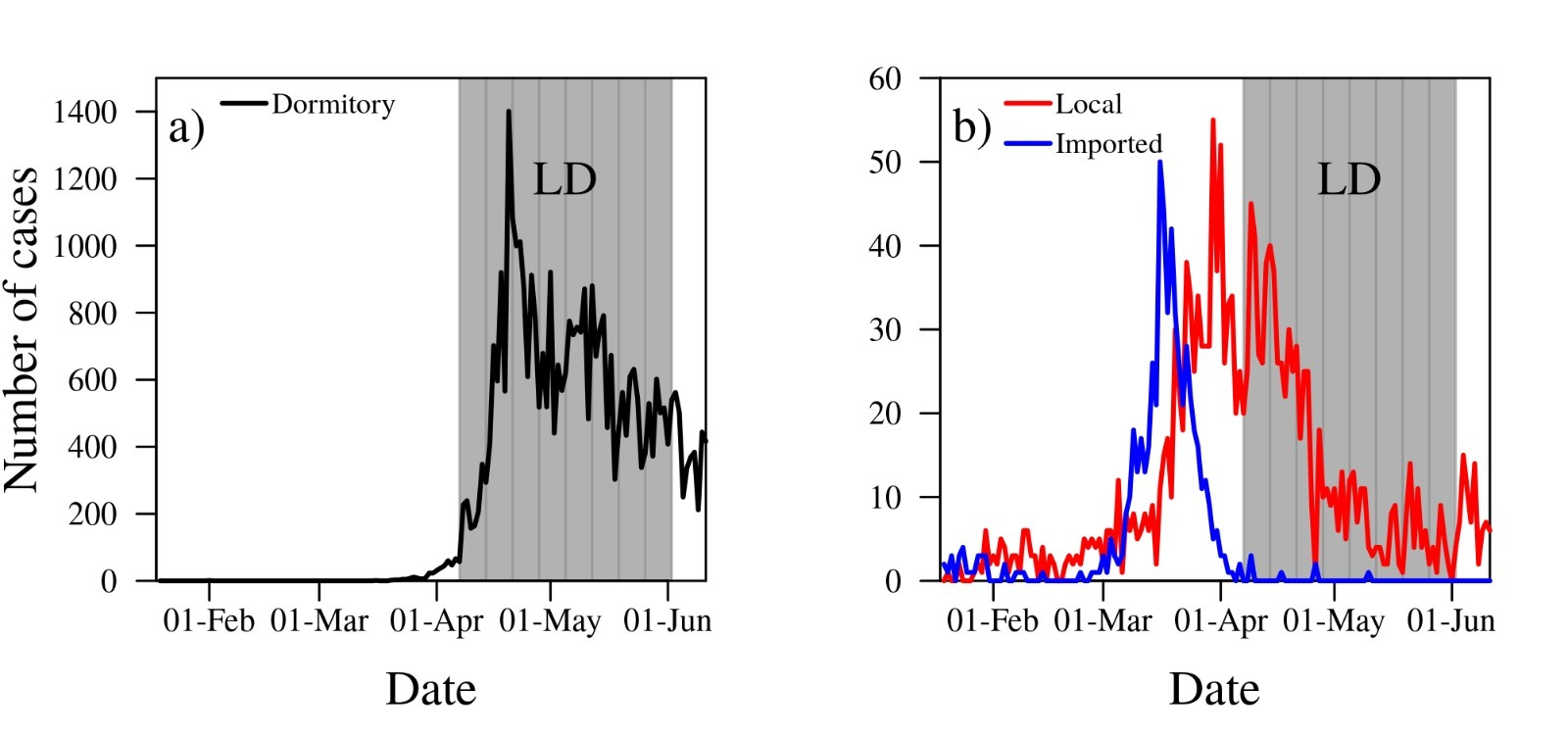


Supplementary Figure 5. Panel a) shows the number of infections occurring in high density dormitory accommodation for foreign workers. Panel b) shows the number of local infections within the resident community and imported infections.

**References**

1. Li, Q. *et al.* Early Transmission Dynamics in Wuhan, China, of Novel Coronavirus–Infected Pneumonia. *N. Engl. J. Med.* (2020) doi:10.1056/NEJMoa2001316.

2. Pung, R. *et al.* Investigation of three clusters of COVID-19 in Singapore: implications for surveillance and response measures. *The Lancet* **395**, 1039–1046 (2020).

3. Mizumoto, K., Kagaya, K., Zarebski, A. & Chowell, G. Estimating the asymptomatic proportion of coronavirus disease 2019 (COVID-19) cases on board the Diamond Princess cruise ship, Yokohama, Japan, 2020. *Eurosurveillance* vol. 25 2000180 (2020).

4. He, X. *et al.* Temporal dynamics in viral shedding and transmissibility of COVID-19. *Nat. Med.* **26**, 672–675 (2020).

5. Chau, N. V. V. *et al.* The natural history and transmission potential of asymptomatic SARS-CoV-2 infection. *Clin. Infect. Dis.* (2020) doi:10.1093/cid/ciaa711.

6. Mossong, J. *et al.* Social Contacts and Mixing Patterns Relevant to the Spread of Infectious Diseases. *PLOS Med.* **5**, e74 (2008).

7. Prem, K., Cook, A. R. & Jit, M. Projecting social contact matrices in 152 countries using contact surveys and demographic data. *PLOS Comput. Biol.* **13**, e1005697 (2017).
